# Letter to the editor in response to Professor Josef Finsterer

**DOI:** 10.1186/s42466-024-00337-0

**Published:** 2024-11-18

**Authors:** Sreelakshmi V, Amrita Pattanaik, Srilatha Marate, Reeta S. Mani, Aparna R. Pai, Chiranjay Mukhopadhyay

**Affiliations:** 1 Manipal Institute of Virology, Manipal Academy of Higher Education, Manipal, Karnataka India; 2 Department of Neurology, Kasturba Medical College, Manipal Academy of Higher Education, Manipal, Karnataka India; 3 Department of Neurovirology, National Institute of Mental Health and Neurosciences (NIMHANS), Bengaluru, Karnataka India

Dear Editor,

We are thankful to Dr. Josef Finsterer for his detailed and thoughtful comments on our recent publication titled, ‘Guillain-Barré syndrome (GBS) with antecedent chikungunya infection: a case report and literature review’. We appreciate the opportunity to address the points raised. To provide further clarity on our findings these are the statements we would like to put forward:

1. **Diagnosis of AMSAN**: The diagnosis of Acute Motor Sensory Axonal Neuropathy (AMSAN) was based on initial nerve conduction studies (NCSs), which showed both axonal and demyelinating features with absent sensory nerve action potentials (SNAPS) in upper and lower limbs. It is known that AMSAN can present with a spectrum of findings, including secondary demyelination in severe cases​. However, considering the predominance of demyelinating features in Chikungunya-related GBS, we agree that AIDP with secondary axonal involvement is a plausible diagnosis​​. Future studies should aim to distinguish these subtypes more clearly. Anti GM1 antibodies were also positive in this case which are more often associated with AMAN/AMSAN [1].
2. **Diagnosis of muscle weakness in an intubated patient**: A screening neurological exam was possible prior to intubation and patient was initially responsive as only mild sedation was used. Paraesthesia was noted based on patient reports post-extubation and from prior to intubation, which were retrospectively documented. This approach, while not ideal, was necessitated by the clinical condition of the patient.
3. **Consideration of Bickerstaff encephalitis**: Bickerstaff encephalitis was considered despite a normal MRI because clinical symptoms such as ophthalmoparesis, facial palsy, and dysphagia strongly suggested this diagnosis. MRI with contrast medium was performed, but no enhancing lesions in the brainstem were detected. These findings do not rule out Bickerstaff encephalitis, as MRI can sometimes be normal in early or mild cases. To support this, there are studies that have concluded that Bickerstaff encephalitis is a clinical diagnosis in many instances [2]. Although GBS can affect cranial nerves altered sensorium is rare and is more often associated with Bickerstaff encephalitis.
4. **Skeletal muscle impairment**: Although skeletal muscle impairment was not prominently featured in the initial case report, subsequent investigations included measurements of creatine kinase (CK), lactate dehydrogenase (LDH), aldolase, and myoglobin, all of which were within normal limits. Needle electromyography (EMG) did not show myopathic changes. Myositis-specific and myositis-associated antibodies were also negative​. These findings effectively rule out significant myositis in this case.
5. **Exclusion of SARS-CoV-2 as a trigger for GBS**: The patient underwent rapid antigen testing for COVID-19 as a part of the ongoing study looking into antecedent infections in Guillain Barre Syndrome and was negative for SARS-CoV-2 antigen. Furthermore, both serum and the CSF samples were negative for anti-SARS-CoV-2 IgM antibodies which ruled out recent COVID-19 infection.

We are grateful for Dr. Finsterer’s thorough review and the opportunity to enhance the accuracy and completeness of our report. This feedback will undoubtedly strengthen the understanding of Chikungunya-related GBS in the medical community.
